# Electron Beam Induced Circularly Polarized Light Emission of Chiral Gold Nanohelices

**DOI:** 10.1021/acsnano.3c09336

**Published:** 2023-11-22

**Authors:** Robin Lingstädt, Fatemeh Davoodi, Kenan Elibol, Masoud Taleb, Hyunah Kwon, Peer Fischer, Nahid Talebi, Peter A. van Aken

**Affiliations:** † Max Planck Institute for Solid State Research, Stuttgart, 70569, Germany; ‡ Institute of Experimental and Applied Physics, Christian Albrechts University, Kiel, 24118, Germany; § Max Planck Institute for Medical Research, Heidelberg, 69120, Germany; ∥ Institute for Molecular Systems Engineering and Advanced Materials, Heidelberg University, Heidelberg, 69120, Germany; ⊥ Kiel Nano, Surface and Interface Science KiNSIS, Christian Albrechts University, Kiel, 24118, Germany

**Keywords:** chiral plasmonics, optical activity, cathodoluminescence, polarimetry, EELS, circular dichroism

## Abstract

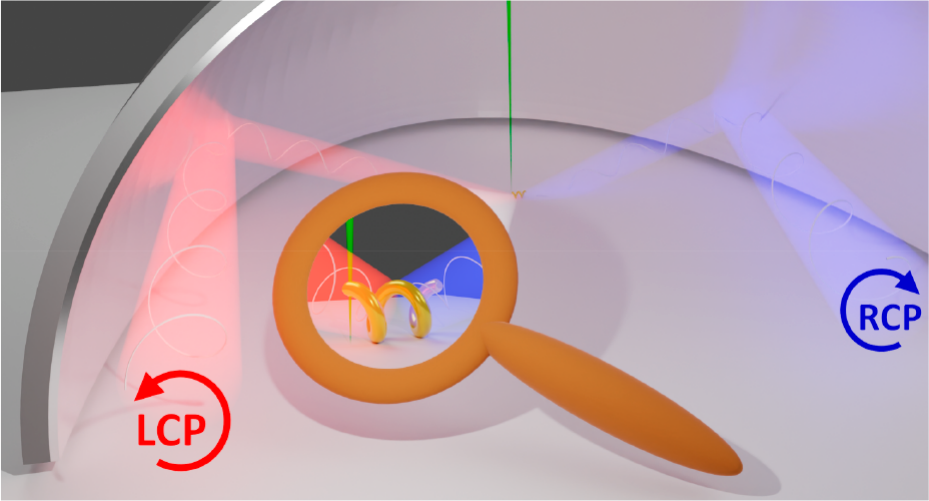

Chiral plasmonic nanostructures possess a chiroptical response orders of magnitude stronger than that of natural biomolecular systems, making them highly promising for a wide range of biochemical, medical, and physical applications. Despite extensive efforts to artificially create and tune the chiroptical properties of chiral nanostructures through compositional and geometrical modifications, a fundamental understanding of their underlying mechanisms remains limited. In this study, we present a comprehensive investigation of individual gold nanohelices by using advanced analytical electron microscopy techniques. Our results, as determined by angle-resolved cathodoluminescence polarimetry measurements, reveal a strong correlation between the circular polarization state of the emitted far-field radiation and the handedness of the chiral nanostructure in terms of both its dominant circularity and directional intensity distribution. Further analyses, including electron energy-loss measurements and numerical simulations, demonstrate that this correlation is driven by longitudinal plasmonic modes that oscillate along the helical windings, much like straight nanorods of equal strength and length. However, due to the three-dimensional shape of the structures, these longitudinal modes induce dipolar transverse modes with charge oscillations along the short axis of the helices for certain resonance energies. Their radiative decay leads to observed emission in the visible range. Our findings provide insight into the radiative properties and underlying mechanisms of chiral plasmonic nanostructures and enable their future development and application in a wide range of fields, such as nano-optics, metamaterials, molecular physics, biochemistry, and, most promising, chiral sensing via plasmonically enhanced chiral optical spectroscopy techniques.

The concept of chirality is a fundamental property that refers to an object’s lack of mirror or inversion symmetry. This results in an object and its mirror image being distinct entities that cannot be transformed through rotational or translational symmetry operations. Despite its seemingly abstract definition, the distinction between these two enantiomorphs is fairly important, and chiral objects are surprisingly present in everyday life. Besides staircases, screws, or human hands, chirality in nature plays an important role down to the nanoscale, as most important biomolecules exhibit exclusively one handedness (homochirality), including essential amino acids and sugars in DNA that form the basis of life.^1,2^ In addition to these geometrical and biochemical considerations, also physical properties are affected and result for instance in chiroptical phenomena; for example, left and right circularly polarized (LCP and RCP) light interacts differently with chiral nano-objects. This effect can be spectroscopically analyzed via circular dichroism (CD) measurements according to
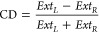
where *Ext*_*L*_ and *Ext*_*R*_ denote the extinction cross sections for LCP and RCP light, respectively.^3,4^ Here, we extend the definition from one that considers only absorption (traditional CD) to one that includes scattering phenomena or radiative losses. A related effect concerns the rotating plane of polarization of linearly polarized light passing through a chiral medium, which is known as optical rotation, and its wavelength dependence as optical rotatory dispersion (ORD).^5^ These techniques are widely used as powerful characterization tools in biological, medical, chemical, or physical applications.^6^ In contrast to most chiral biomolecules or natural media, which only show a comparably weak chiroptical response, this effect is increased by several orders of magnitude for chiral plasmonic nanostructures or metamaterials, where electrons can be collectively excited to perform harmonic oscillations at the surfaces.^7^ As a consequence, major efforts have been made to create artificial chiral nanostructures via various methods^8−15^ and characterize them through chiroptical measurements.^16^ As an example for three-dimensional geometrically chiral structures, gold nanohelices have been fabricated in a top-down approach to study the influence of structural variations such as pitch or helical radius on chiroptical effects.^17,18^ A shift of the response in the dichroic signal from the infrared to the visible spectral range could be achieved for even smaller helices, produced via an alternative shadow-growth technique, following a bottom-up approach.^19,20^ Optical CD measurements that revealed strong chiroptical effects were performed on ensembles but also on freely diffusing single nanohelices,^21^ as well as on other single nanocrystals and fabricated nanostructures.^22^ Consequently, the investigation of individual chiral plasmonic structures with high spatial resolution, as provided by electron microscopy, gives additional insight, especially as the aforementioned localized surface plasmon resonances (LSPRs) can be excited by electron beams in a wide energy range and analyzed via electron energy-loss spectroscopy (EELS).^23,24,33,34,25−32^ Furthermore, the generated cathodoluminescence (CL) radiation^35,36^ carries valuable polarization information, also in relation to the chirality of the underlying structure.^37−39^ In contrast to previous works, where these analytical techniques were mainly applied to arrays or individual chiral nanostructures with a planar geometry, in the present study, individual three-dimensional chiral gold nanohelices are investigated with high spatial resolution inside transmission (TEM) and scanning (SEM) electron microscopes. The combination of CL polarimetry and EELS is used to measure angle-resolved far-field radiation patterns of left- and right-handed structures and unravel the underlying plasmonic modes. Numerical simulations reveal that the observed CL emission in the visible spectral range originates from the decay of transverse dipolar modes that arise from the three-dimensional helical shape. We show that excited nanohelices emit directional circularly polarized light (CPL) and that its circular polarization state is correlated with both the handedness of the structure and the electron beam position.

## Results

### Optical and Structural Characterization

The fabrication of chiral gold nanostructures was achieved through a combination of block copolymer micelle lithography (BCML) and glancing angle deposition (GLAD). This nanoGLAD process is described in more detail in the Methods section and illustrated in Supplementary Figure S1. It allowed for the creation of helical nanostructures with a height of approximately 240 nm and an outer diameter of 140 nm. The pitch between two windings is 110 nm, with a thickness ranging from 20 to 40 nm. Importantly, both left- and right-handed helices were successfully synthesized by selecting the corresponding rotation direction during the growth.

**Figure 1:**
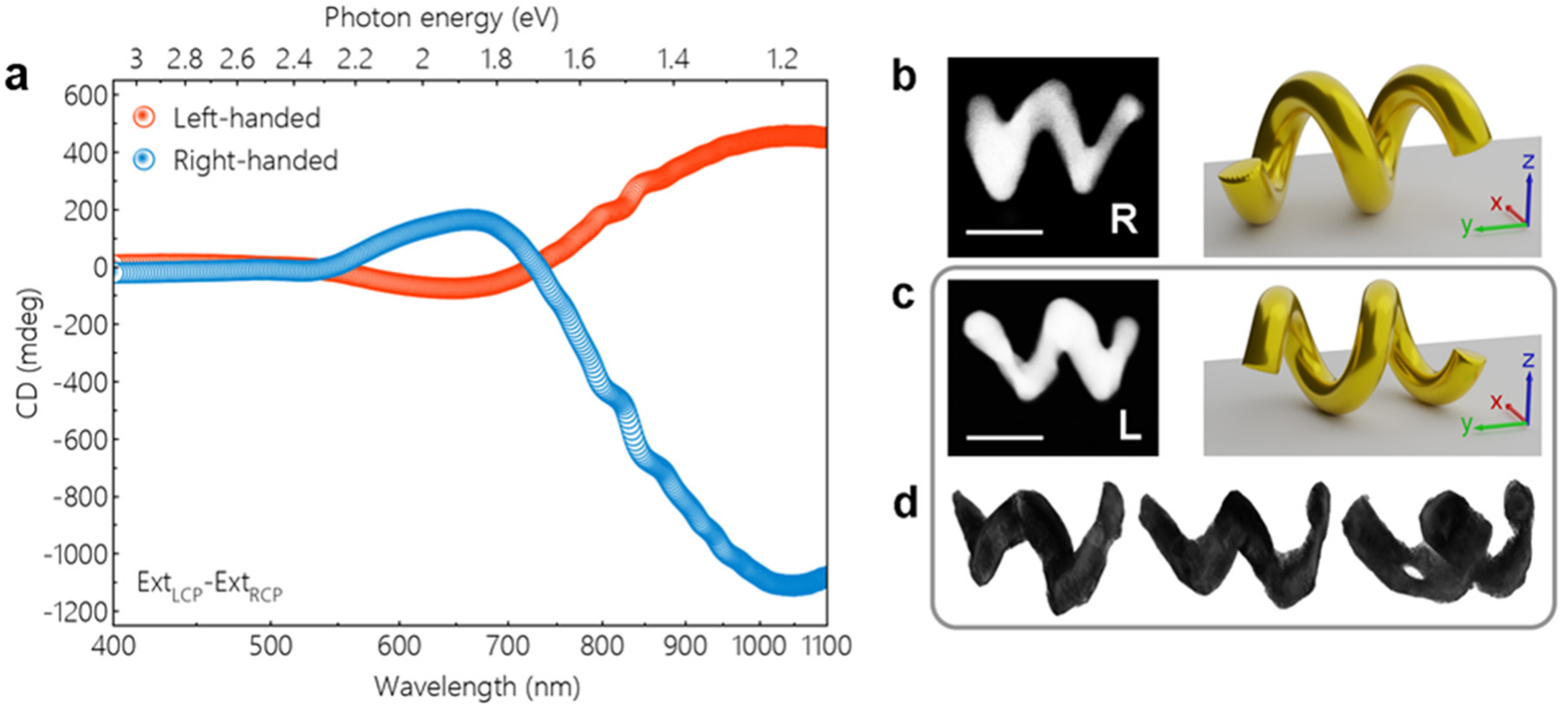
Optical activity and geometrical characterization. (a) CD measurements in solution revealing the optical activity of the fabricated chiral nanostructures. The extinction of LCP and RCP light is related to the handedness of the measured structures. (b, c) Individual nanohelices were geometrically characterized by STEM techniques. HAADF images show two-dimensional projections for a right-handed helix (R) and a left-handed one (L). The corresponding models of the three-dimensional structures and their orientation were determined by perspective mapping. (d) Tomographic reconstruction of the left-handed helix reveals its handedness via the observed helical shape. The structure is shown for different orientations. The reconstruction of a right-handed helix is shown in Supplementary Figure S2. Movies of the rotating three-dimensional models are available online. The scale bars are 100 nm.

CD extinction measurements were conducted in metafluids containing helices with different handedness, revealing their optical activity. As shown in Figure 1a, the relative extinction strength for LCP and RCP light correlates with the handedness of the chiral structure. The change in the sign of the CD signal, commonly referred to as the Cotton effect, has been reported for similar nanohelices^19^ and other plasmonic chiral nanostructures.^12,40,41^ In comparison to the aforementioned previous studies on gold nanohelices with smaller dimensions, the overall dichroic response of the helices produced in this study was found to be red-shifted toward longer wavelengths in the spectrum.

Individual chiral gold nanohelices were investigated further by means of analytical TEM, regarding both their geometrical and inherent material properties. High-angle annular dark-field (HAADF) images were acquired with a scanning transmission electron microscope (STEM) for right- and left-handed structures (Figure 1b and c). From these two-dimensional projections, some of the geometrical properties like helix radius, number of windings, and their thickness can be measured directly. For a full geometrical characterization, however, the three-dimensional shape of the structures and their orientation on the flat amorphous carbon substrate must be considered. Perspective mapping of constructed model helices revealed a flat orientation with a rotation around the long axis, as shown by the illustrations next to the STEM images. The created models resemble the real helices in an idealized and hence simplified form and are used as a basis for theoretical calculations and simulations. A qualitative confirmation that the experimentally grown helices indeed show the expected handedness was achieved by electron tomography (Figure 1d). Images of the structure of interest are acquired under various tilt angles over a wide angular range and spatially aligned by cross-correlation filtering. A three-dimensional volume of the sample is then reconstructed from this data set by applying the weighted backprojection (WBP) algorithm. The subsequently applied simultaneous iterative reconstruction technique (SIRT) optimizes this initial model further by continuously comparing slices of the reconstructed volume with the original images of the tilt series.^42^

### LSPRs of Gold Nanohelices

**Figure 2:**
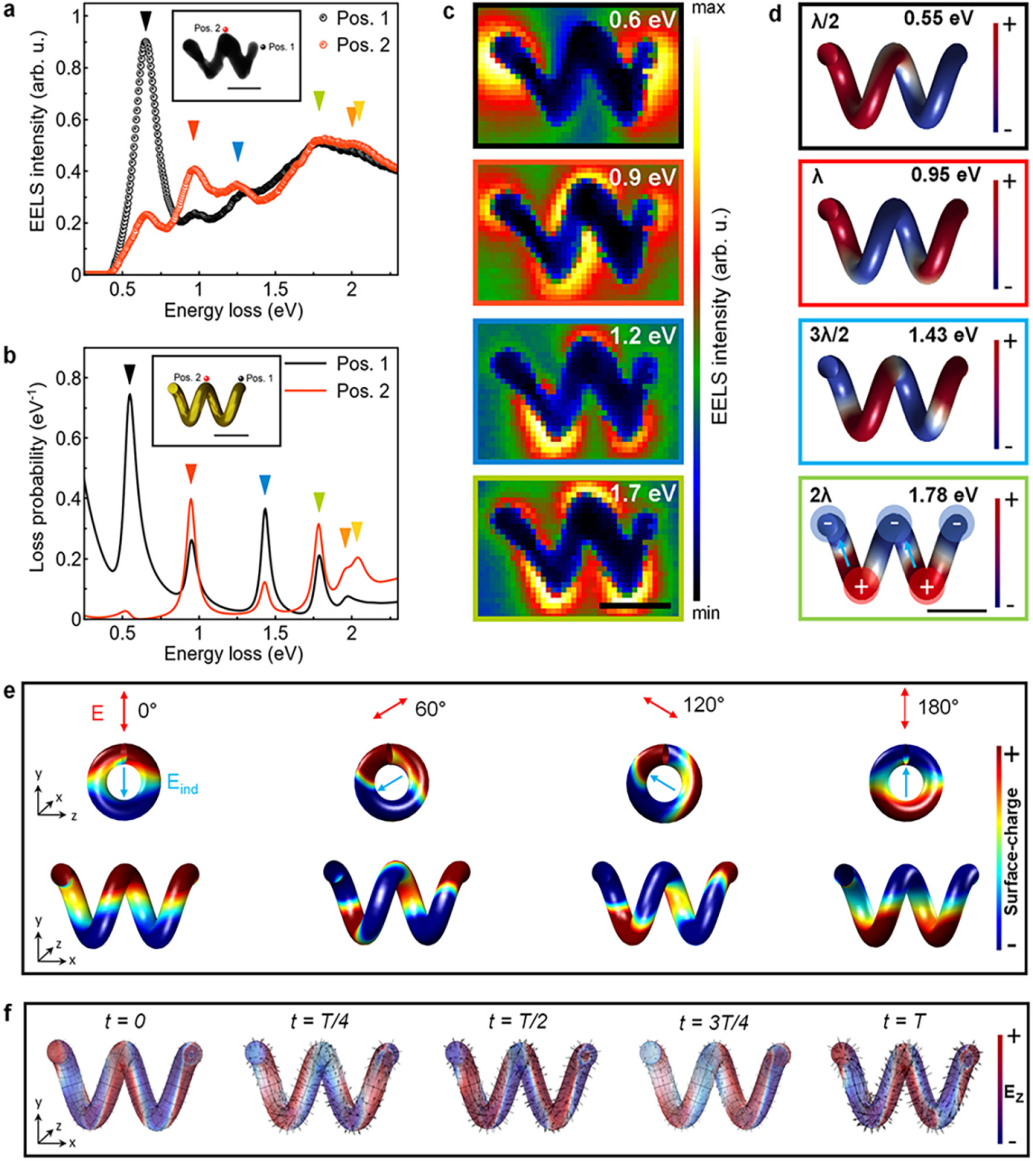
LSPRs of gold nanohelices. (a) Experimental EEL spectra of a left-handed nanohelix being excited at positions 1 at the end of the structure (black curve) and 2 at the center winding (red curve), as marked in the inset graphic. A strong spectral peak at the lowest resonance energy of around 0.6 eV is observed at the end of the structure, whereas the second resonance of around 0.9 eV is stronger at the center. (b) Simulated EEL spectra obtained via the BEM. (c) Energy-filtered images at the indicated resonance energies over a width of 0.1 eV, mapping the spatial EELS probability and emphasizing the character of multiple-order plasmonic resonances. (d) Simulated plasmonic eigenmodes computed for the depicted resonance energies. Although the longitudinal plasmonic modes follow the helical windings, a secondary charge oscillation forms due to the three-dimensional geometry of the structure. The scale bars are 100 nm. (e) Simulated surface-charge distribution for a left-handed helix, being excited through an electric field, oscillating in the plane perpendicular to the long axis of the helix. (f) Dynamic simulation of plasmon excitations within the helical structure that cause a rotating electric field in three dimensions (arrows). The vertical electric field component *E*_*Z*_ is mapped via the red and blue color code. The evolution over a full cycle is more visible in the dynamic representation, which is available online.

The plasmonic properties of individual gold nanostructures were investigated via EELS measurements that were conducted using the ZEISS SESAM microscope, a monochromatic STEM. This instrument provides both high energy and spatial resolution, making it ideal for low-loss experiments (see Methods for details). The EELS results provide insights into the projected photonic local density of states (PLDOS) over a wide energy range^43^ and allow for mapping the spectral and spatial distribution of surface and localized plasmon polaritons.^44,45^ By acquiring spectral intensity variations at selected electron impact positions, the technique reveals localized plasmonic resonances, which are strongly dependent on the sample geometry. For gold, this spectral information is accessible up to the interband transition threshold of about 2.38 eV,^46−48^ beyond which interband absorption results in a uniform intensity distribution.^49^ To obtain spatially resolved spectral information about the localized plasmons, spectrum images were acquired with a spatial sampling of 7 nm, providing a three-dimensional data cube, where each voxel contains a low-loss spectrum with an energy resolution of approximately 80 meV, as determined by the full width at half-maximum (fwhm) of the zero-loss peak (ZLP). The EELS findings are confirmed through simulations based on the boundary element method (BEM),^50,51^ which solve Maxwell’s equations with respect to the real three-dimensional shape of the sample. The distinct architecture of the helix comprises two full windings with a consistent pitch of 105 nm and a clearly defined outer helical diameter of 135 nm. Its thickness was precisely measured, with an average value of 36 nm in diameter. These dimensions were crucial for subsequent BEM simulations of the helix’s material properties and plasmonic resonance effects. The experimentally acquired and simulated electron energy-loss (EEL) spectra for the left-handed helix are depicted in Figure 2a and b, respectively. The spectroscopic data for a right-handed structure can be found in Supplementary Figure S3.

Spectra were extracted at the most significant excitation positions at the end of the helix (position 1, black line) and at the center winding (position 2, red line), both in an aloof configuration, i.e., passing by the specimen in close proximity, as marked in the inset graphics. The experimental signal-to-noise ratio (SNR) was improved by averaging over four neighboring pixels. Overall, the experimental and simulated results agree well with only slight deviations. The observed discrepancy in the resonance energies between experimental and numerical EEL spectra is attributed to the mismatch between the actual experimental structure and the model, mainly due to the surface roughness and limitations in extracting the exact configurations via tomography. The experimental structure differs from the model, featuring a helix with inclined edges, a rough surface, and a varying thickness distribution.

In Figure 2a and b, the pronounced spectral peak at the lowest energy around 0.6 eV (black triangles) is mainly present at excitation position 1 with a redshift of only 100 meV in the simulated spectrum. Both data sets show the highest excitation efficiency for the second resonance at around 0.9 eV (red triangles) close to the center winding at position 2. The third resonance at 1.25 eV (blue triangles) is found to be blue-shifted at 1.43 eV in the simulation, with a discernible decrease in intensity near the center winding. Both experimental and simulated data show a fourth resonance around 1.8 eV (green triangles), with the experimental data presenting a less distinct peak. A shallow maximum in the experimental spectrum around 2 eV, mainly visible at position 2, is associated with the fifth and sixth resonances, which are close in energy in the simulation (orange and yellow triangles).

In a comparison of a helical nanostructure with a straight nanorod of equivalent strength and length (Supplementary Figure S4), we observe similarities in the resonance behavior. Both systems exhibit “antenna modes”^52^ that have been reported also for silver helices.^53^ However, there are also notable differences, such as all higher-order resonances in the simpler straight rod geometry being red-shifted at lower energies, due to the lack of interwinding interactions. As illustrated in Figure S4, both a left-handed helix and a straight nanorod of the same size exhibit plasmon modes with slightly different energies. The computed eigenmodes (first four modes in Figure S4) reveal that both structures possess similar plasmon modes with slight energy differences. Notably, the plasmon modes of the helix are observed at slightly higher energies compared to those of the nanorod. The main reason for such a difference is the three-dimensional configuration of the helix that allows for the excitation of a rotating transverse electric field, as well as a transverse dipolar excitation. Thus, we ascertain that this discrepancy primarily arises from mode mixing induced by the closely spaced windings of the helix. For the same reason, the selection rule that the lowest-, third-, and fifth-order modes are not excitable at the center of a rod (position 2) is weakened and, even more intriguingly, reversed for higher-order resonances, as transverse dipolar charge oscillations arise for the three-dimensional helical geometry. These findings suggest that interwinding interactions play a significant role in determining the excitation behavior of the helical nanostructures.

The spectra exhibit weaker resonances at higher energies. However, the spatially resolved EELS probability maps, extracted over a 100 meV range at the depicted resonance energies (Figure 2c), showcase the characteristic pattern of dipolar and multiple-order LSPRs, a well-known phenomenon supported by previous theoretical studies and experimental observations, for example in gold nanorods.^54−62^ Furthermore, the relation between the lowest (dipolar) resonance energy and the real three-dimensional path length of each investigated helical structure aligns with the values determined for gold nanorods through theoretical and experimental investigations (Supplementary Figure S5).

The validity of the hypothesis that charge oscillations occur along the helically twisted rod is substantiated by simulated surface-charge distributions and plasmonic eigenmodes computed for the specified resonance energies, as marked in Figure 2d. Plasmonic modes remain clearly visible for a more general excitation position (Figure S6). The plasmonic eigenmodes are labeled with the relative path length of the helical structure in comparison to the corresponding wavelength, λ, at the resonance energy. The simulations reveal a consistent pattern of longitudinal multiple-order LSPRs along the helical windings, with only a localized perturbation observed at the excitation position. This provides strong evidence for the occurrence of charge oscillations along the helically twisted rod. In addition, the 2λ- and higher-order plasmonic eigenmodes result in transverse dipolar LSPRs oriented along the short axis of the nanohelices. In other words, in addition to the longitudinal dipole–dipole interactions due to Fabry–Pérot-like resonances along the windings, the helical geometry leads to secondary charge oscillations with dipolar character, as illustrated in Figure 2d. A similar transverse dipole distribution has been reported for nickel and silver nanohelices.^63^ Numerical simulations revealed that the transverse dipole can be excited through an electric field oscillating in the plane perpendicular to the long axis of the helix (Figure 2e). The orientation of the induced surface-charge distribution coincides with the polarization direction of the driving field. Notably, the electron moving along a direction perpendicular to the long axis of the helix couples predominantly to the component of the electric field along the same direction as well.

Dynamic simulations revealed that plasmon excitations within the helical structure lead to the formation of a rotating transverse-electric wave. The propagation direction of this rotating wave away from the excitation position induces the radiation of CPL in the far field with matching handedness and primarily oriented along the long axis of the helical structure. This dynamic process is illustrated in Figure 2f. A time-resolved movie of electric field evolution is available online. Reflection from the end of the metallic structure leads to CPL emission in the opposite direction with inverted relative handedness and reduced intensity due to absorption effects. Light emitted to the normal direction with respect to the surface is canceled, due to the prominent transversally oriented dipolar response of the structure (Figures S7 and S8).

### Angle-Resolved CL Polarimetry

**Figure 3:**
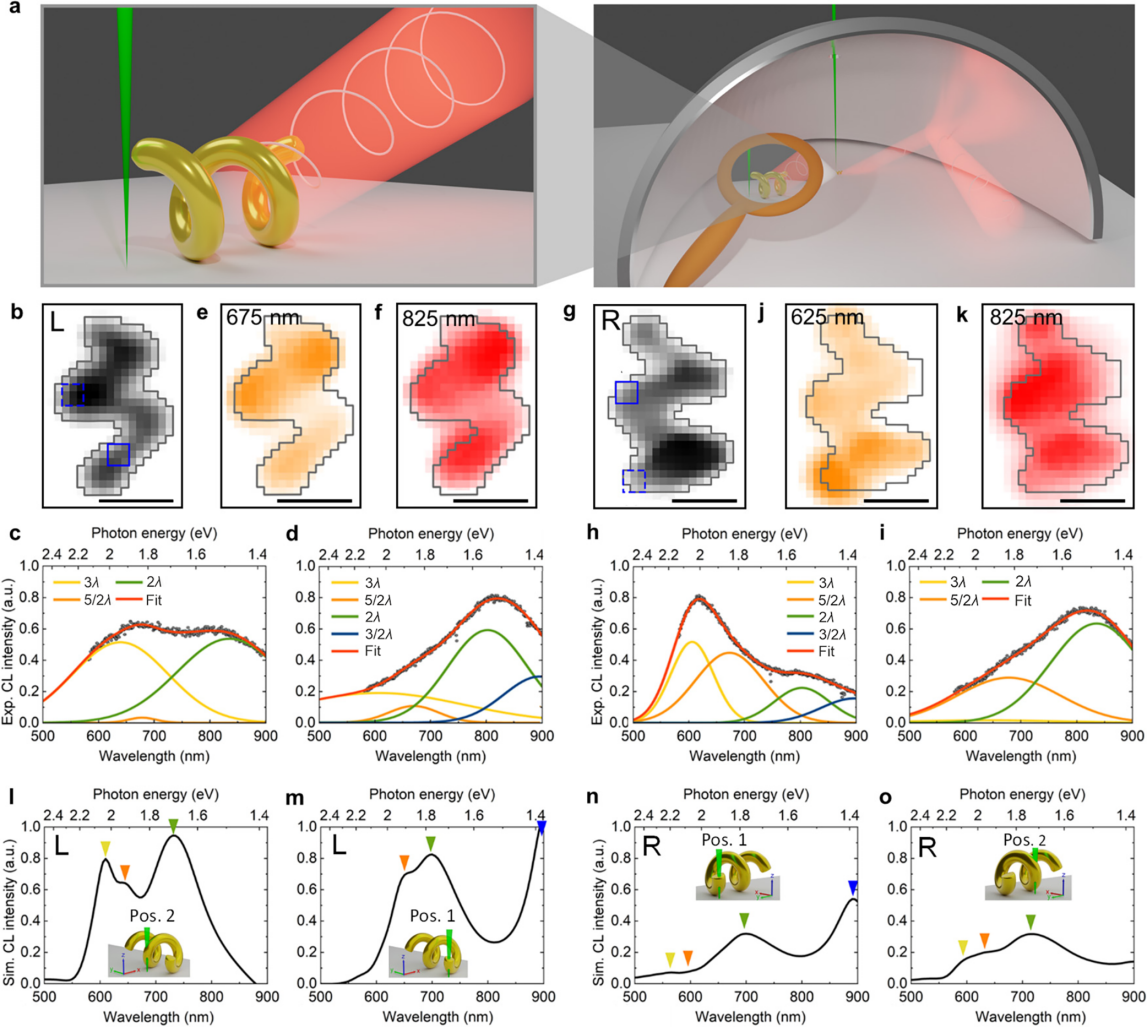
CL spectroscopy. (a) Schematic of the experimental setup. The nanostructure is excited by an electron beam that passes through a hole in the parabolic mirror. The specimen, precisely positioned in the focal point of the parabolic mirror, emits light that is transformed to a parallel beam by the mirror and directed outside the microscope for in-depth analysis. (b, g) Secondary electron images were used to determine the circumference (gray line) of the left (L) and right (R) structures. (c, d, h, i) CL spectra extracted from the positions marked on panels b and g. Strongest emission is observed between 600 and 700 nm and above 800 nm. (e, f, j, k) Spatially resolved excitation efficiency maps are shown for the indicated center wavelength with a bandwidth of 50 nm. The scale bars are 100 nm. (l–o) Simulated CL spectra in the visible range reveal emission maxima that are related to plasmonic resonance energies (Figure 2) and marked with triangles in corresponding colors. Insets show the three-dimensional models of the left-handed and right-handed nanohelices, being excited at different positions by the electron beam (illustrated in green color).

The optical activity of an ensemble of gold nanohelices was previously confirmed by CD measurements. Here, we further explore the properties of the light emitted upon electron impact for individual nanohelices at the nanoscale by CL spectroscopy techniques within an SEM. While raster scanning a focused electron beam over the area of interest, multiple signals including secondary electrons (SEs) for imaging and the emitted cathodoluminescence light are detected and analyzed. The far-field radiation of the nanostructure, excited by the electron beam, is collected by a parabolic mirror, as shown in Figure 3a, and directed outside the microscope for further analysis.

In the spectroscopic imaging mode, emission spectra are measured for each spatial pixel, with the highest intensity recorded for wavelengths between 600 and 700 nm and above 800 nm, as shown in Figure 3b–k. The correlation between electron beam position and the emission intensity, filtered for these wavelengths, results in a set of spatially resolved excitation efficiency maps, displayed in corresponding colors in arbitrary units. The geometrical orientation of the structures does not significantly affect the excitation process, as confirmed by measurements of a tilted helix (Figure S9). The intensity distribution reveals five key positions, the two ends and the three most extended points along the helical windings. This information is crucial to the subsequent angular and polarimetric measurements that are beneficially acquired at the positions demonstrating the highest excitation efficiency and spectrally filtered around the peaks of the CL spectra. We note that the higher-order plasmonic modes 5/2 λ and 3λ (orange and yellow triangles in Figure 2), which correspond to radiation around 600 nm, are expected to be nonradiative in CL experiments.^59^ However, due to the three-dimensional shape of the structure, secondary transverse dipolar charge oscillations form and their decay results in the observed radiation, making the otherwise dark modes bright upon electron excitation at specific positions of the nanohelix.

According to theoretical predictions, based on a separate discussion of the full and radiative electromagnetic local density of states (EMLDOS) of metallic nanoparticles, at the length scales discussed here, a direct link can be assumed between the resonance energies in EELS and the intense far-field radiation in CL measurements at corresponding wavelengths.^59,64^ The emission in the visible spectral range from the investigated helical nanostructures is attributed to plasmonic modes with resonance energies between 1.4 and 2 eV. Meanwhile, lower-order modes radiate in the infrared, which cannot be detected by the spectrometer used in this study. To validate these findings, numerical calculations were conducted using idealized three-dimensional models, consistent with those used in the EELS analysis, to predict the emitted far-field radiation from individual left- and right-handed nanohelices at characteristic excitation positions (Figure 3l–o).

In this study, we carefully compared emission peak positions in the simulated CL spectra (Figure 3l–o) with measured and simulated resonance energies in EELS (Figure 2a and b), paying special attention to the spectral shape and excitation efficiency in relation to the excitation position. Our findings indicate that for the left-handed helix the enhanced emission around the wavelengths 900, 730, 640, and 610 nm corresponds to plasmonic resonances at 1.43 1.78, 1.98, and 2.04 eV, respectively, which are marked with triangles in the corresponding colors blue, green, orange, and yellow. In particular, the highest-order mode 3λ, marked with a yellow triangle, radiates more strongly around 610 nm than the neighboring one 5/2 λ (orange triangle) around 640 nm. However, the former is exclusively excitable at the central winding (position 2). Conversely, the radiation around 900 nm (blue triangle) only occurs when the helix is excited at the end (position 1), which is represented in the simulated energy-loss probability of the corresponding resonance of 3/2 λ at 1.43 eV as well (Figure 2b). The most dominant 2λ mode, which most likely corresponds to the one observed in the CL experiment (Figure 3b–f), radiates around 730 nm (green triangle) and is excitable at both the end and center winding of the helix. The spectral features in the experiment do appear slightly red-shifted at longer wavelengths. However, these deviations can again be attributed to the simplified geometrical assumptions made in the simulation environment and the used dielectric function. The simulated CL spectra for the right-handed structure (Figure 3n,o) exhibit similar spectral features. In the collective agreement of measured and simulated EELS and CL spectra, the plasmonic resonances appear blue-shifted at higher energies and shorter wavelengths, as compared to the left-handed structure, due to its slightly different geometry. Although both structures have the same number of turns, there are variations in their radii and pitch. The left-handed helix in our study has a diameter of 36 nm and a pitch of 105 nm, while the right-handed helix is larger in all dimensions with a diameter of 44 nm and a pitch of 115 nm. These variations contribute to the observed differences in their CL spectra.

**Figure 4:**
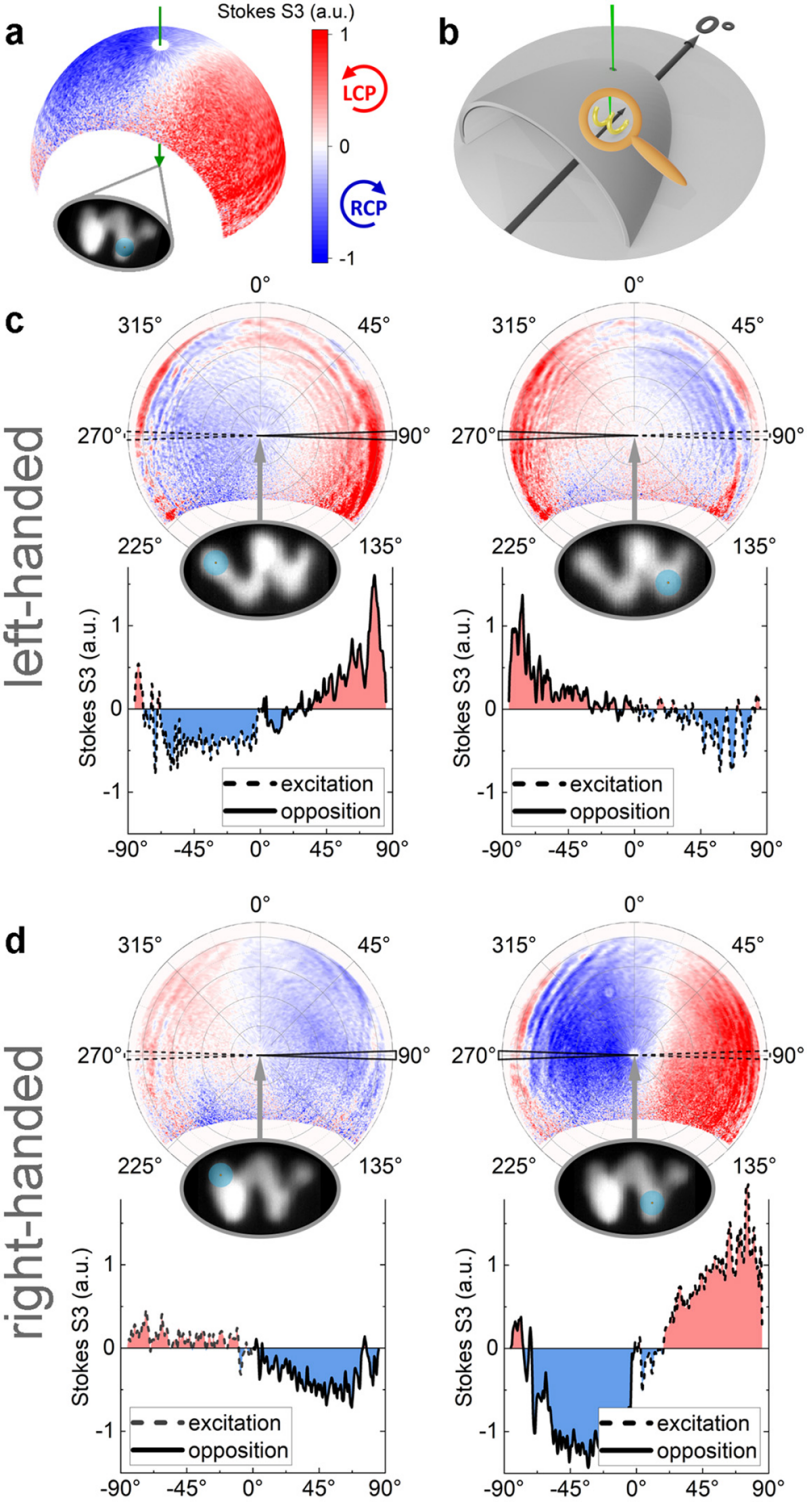
Angle-resolved CL polarimetry. (a) Three-dimensional plot of an angle-resolved far-field intensity pattern on a fictional hemispheric detection plane. The light is emitted by the nanoscopic sample in its center, excited by the electron beam at the position marked in the inset graphic. The color code shows the normalized differential signal between the emission intensity of left (red) and right (blue) circularly polarized light (Stokes *S*3) for a wavelength of 800 nm and is valid for all subsequent spherical polar plots. (b) The helices are oriented physically with their long axis perpendicular to the main axis of the parabolic mirror (black arrow). (c, d) Two-dimensional plots of the same dichroic signal like in panel a for nanohelices with different handedness. When exciting at the ends of a left-handed structure (c), relatively more LCP light is emitted in the opposite direction, which is the “pointing-direction” of the helix. This effect is visualized through line plots along the long axis of the helices. The same effect, but with reversed circular polarization, is observed for a right-handed helix (d).

In contrast to the aforementioned spectroscopic imaging mode, where the far-field radiation is accumulated over the entire accessible angular range, the separation of radiation directions in the angle-resolved configuration adds multidimensionality to the measured signal itself. In combination with band-pass and polarizing filters, the emitted light of an excited structure can be characterized in great detail. The chiral nature of the nanostructures under investigation requires a particular focus on their circularly polarized components. The normalized differential signal between the left (red color) and right (blue color) circularly polarized emission defines the normalized Stokes parameter *S*3 according to
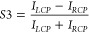
and provides insight into the directional emission properties concerning these polarization states (Figure 4a). The position of the actual excitation is marked in the inset graphic.

An angle-resolved far-field intensity pattern projected onto a hemispherical detection plane is shown in Figure 4a. Figure 4b displays the orientation of the helices with respect to the main axis of the parabolic mirror. The structure is located in the center and oriented as shown in the inset graphics, along with the marked excitation position. A quantitative two-dimensional representation of the hemispherical intensity distribution is given by spherical polar plots (Figure 4c and d), with the polar angle on the radial axis and the azimuthal angle clockwise along the circumference. Analyzing the angle-resolved differential signal between LCP and RCP light intensities reveals two intriguing effects consistent over all investigated structures. The first is that the strongest effect is observed in the direction of the long axis of the helical structure for polar angles approaching 90° and toward the opposite side of the excitation position at one end of the helix. The second reason is that the dominating handedness of the circularly polarized light emitted in this direction coincides with the handedness of the chiral structure itself. Line scans following the polar angle over the hemisphere in a parallel orientation to the long axis emphasize this effect. The differential signal in the excitation direction (dashed line) and in the opposite direction (solid line) is plotted below each graph, with the same behavior observed in the reversed circular polarization for right-handed helices (Figure 4d). For excitation positions at the center winding (position 2) no such consistent effect is observed.

**Figure 5:**
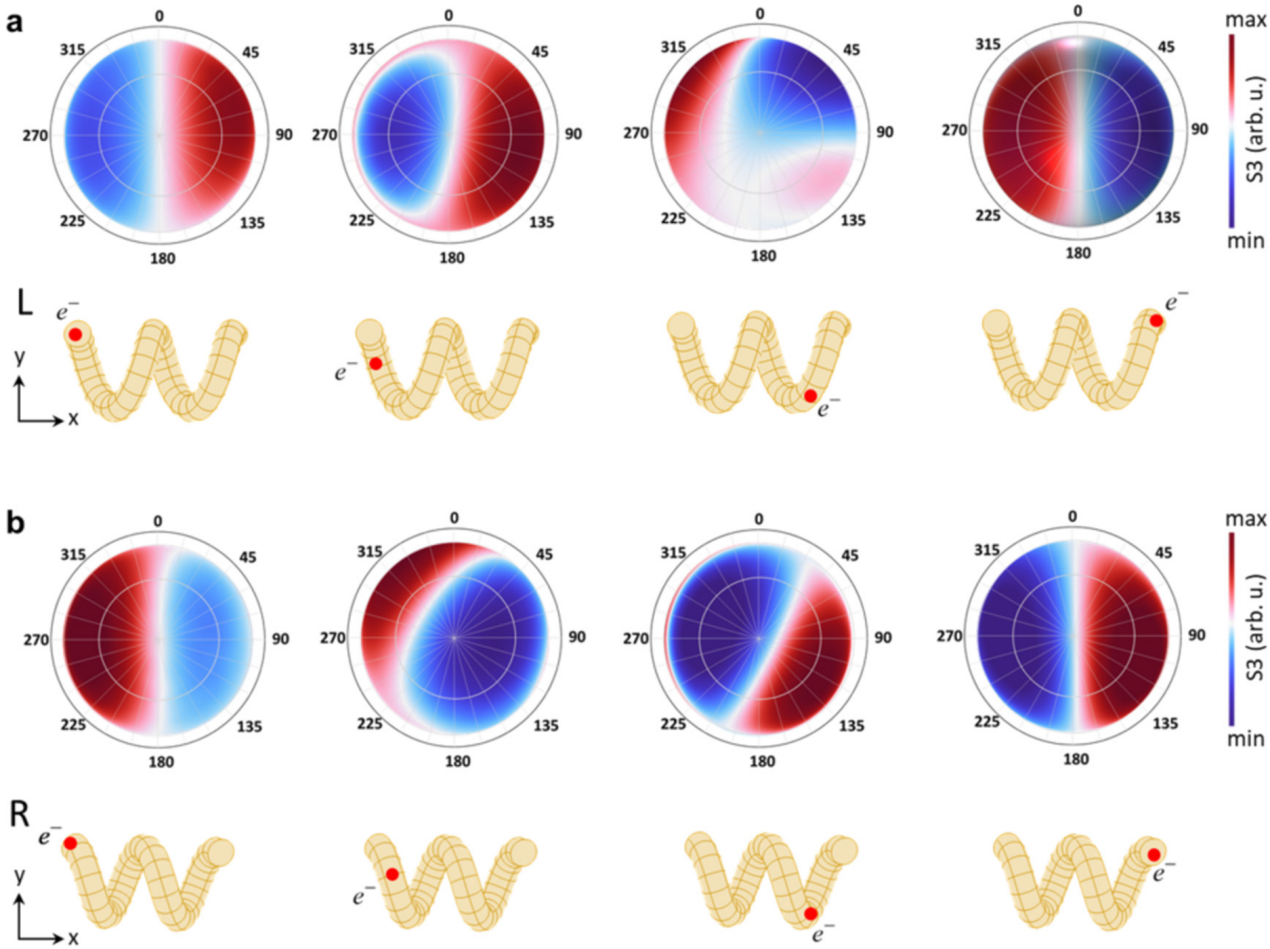
Angle-resolved circular polarimetry simulations. Spherical polar plots of the simulated angle-resolved Stokes parameter *S*3 for the left-handed (a) and right-handed nanohelix (b) show whether more LCP or RCP light is emitted in certain directions. The excitation positions by the electron beam are marked on the three-dimensional models below each plot. The excitation position 2 at the center winding is not included here, as spectral simulations revealed no difference in the dichroic signal, as shown in Figure S11.

In order to provide additional evidence for our experimental findings, we performed angle-resolved circular polarimetry simulations for both left-handed and right-handed helices excited at various positions. The resulting spherical plots, as displayed in Figure 5, demonstrate good agreement with the experimental angle-resolved intensity distributions of the dichroic signals (Figure 4). Furthermore, the radiation emitted toward the top and bottom hemispheres results in similar patterns, as it is exemplarily shown for a right-handed helix in Figure S10. As shown in Figure S11, for the extreme excitation positions at the ends of the helices (Pos. 1), a qualitative inversion of the sequence of maxima and minima in the investigated spectral range is observed. For excitation position 2 at the center winding, the dichroic signals are nearly identical.

## Conclusion

Our study demonstrates the successful preparation and optical characterization of the chiroptical response of both left- and right-handed chiral gold nanohelices. The shape of individual particles and their handedness were confirmed as expected through electron tomography reconstructions. We performed a comprehensive investigation of plasmonic resonances using a combination of EELS measurements and numerical simulations. Our results show that multiple-order longitudinal LSPRs are related to surface-charge oscillations along the helical windings, while interwinding interactions lead to slightly shifted resonance energies compared to straight rods with similar thicknesses and (path) length. In addition, higher-order modes induce the formation of secondary transverse dipolar LSPRs oriented along the short axis of the nanohelices. The radiative decay of these modes results in the observed CL emission at the corresponding wavelengths. Angle-resolved CL polarimetry measurements of the emitted far-field radiation revealed that the emission characteristic of circularly polarized light is related to the handedness of the chiral structure and depends on the excitation position. Our simulations of the far-field radiation of excited nanostructures further confirmed that the peaks in the CL intensity correspond to plasmonic resonances. Furthermore, both the excitation efficiency of these modes and the circular polarization characteristic of the emitted light strongly depend on the position of the exciting electron beam, the chirality of the structure, and other geometrical properties. Studies of chirality for single nanostructures depend not only on the handedness of the structure but also on the relative orientations of the structure and the excitation and detection beams. Here, electron beam induced methods are particularly powerful in comparison with purely optical methods, as the exact geometric properties of the structure and the precise orientations can be determined together with the corresponding spectral response. The high precision facilitates the comparison with theory and suggests that electron beam single chiral particle analysis can be a powerful tool to analyze chirality at the smallest of scales. Especially, the combined study via EELS and CL polarimetry is a promising technique in this regard. The complex interplay between exciting electrons, plasmonic modes of three-dimensional structures, and the resulting far-field radiation provides exciting opportunities for tailoring the chiroptical properties of nanostructures, with potential applications in a variety of scientific and technological fields. Further in-depth investigation is necessary to fully understand and exploit this promising area.

## Methods

### Sample Fabrication

Gold nanohelices were fabricated through a combination of nanopatterning and GLAD as described previously.^19,20^ A silicon wafer was prepared with a monolayer of hexagonally arranged gold nanoparticles by using block copolymer micelle lithography (BCML). This seed pattern for the following growth process contained particles that were 17 nm in size with a nearest-neighbor distance of 120 nm. During the growth, gold and copper (Au_0.95_Cu_0.05_) were co-deposited via electron beam induced physical vapor deposition (PVD) with a constant rate of 0.4 nm s^–1^. The sample surface was cooled with liquid nitrogen to 90 K and slowly rotated around its normal axis while keeping a constant angle of 87° with respect to the material flux direction.

### Optical Characterization

CD measurements were performed with a Jasco J-810 CD spectrometer as reported in previous work.^19^ Gold nanohelices were sonicated off a cut substrate into 1 mL of purified Milli-Q water. Assuming a hexagonal array of nanoparticles, the colloidal concentration is around 1.85 × 10^9^ mL^–1^. Left- and right-handed helices were measured separately.

### Sample Preparation

For analytical electron microscopy investigations, either left- or right-handed nanohelices were sonicated off of wafer pieces into purified water and deposited via drop-casting onto the amorphous carbon membrane of mesh-200 copper TEM grids. Prior to electron energy-loss measurements under long and intense electron beam exposure, the sample grid was slowly heated to 400 °C in a vacuum to remove any residual contamination due to mobile hydrocarbons and cooled again to room temperature.

### Electron Tomography

Images of left- and right-handed nanohelices were acquired in conventional TEM mode as a tilt series in 1° steps over the accessible angular range from +77° to −58° and +84° to −66°, respectively. Imaging was performed in conventional TEM mode with a JEOL ARM-200F electron microscope operated at 200 kV. The TEM grids were cut in an orientation to enable the maximum accessible tilt range before shadowing and glued onto the grid support rod of the GATAN model 912 dedicated tomography holder. The acquired image stacks were spatially aligned by cross-correlation filtering and used to reconstruct the three-dimensional shape of the structure by applying a combination of the WBP algorithm and the SIRT.

### EELS Measurements

EELS were performed using the ZEISS sub-electronvolt-sub-angstrom microscope (SESAM).^65^ This instrument is ideally suited for low-loss investigations because of its high energy dispersion at a good stability and its high energy resolution. Electrons are extracted from a Schottky field emission gun and pass through an electrostatic Ω-type monochromator before they are accelerated by 200 kV. In combination with the in-column MANDOLINE energy filter, an energy resolution of around 70 meV can be achieved, measured as fwhm of the ZLP in vacuum. Energy-loss measurements were performed as spectrum images, where spectra are measured for each spatial pixel while the focused electron probe is raster scanned over the area of interest. From this three-dimensional data cube, resonance energies can be determined, and spatially resolved EELS probability maps can be extracted by filtering for certain energy-loss ranges. Individual spectra were aligned with respect to the maximum of the ZLP and filtered with principle component analysis before the background was subtracted by fitting a power-law function.

### EELS Simulations

Numerical calculations of electron energy-loss spectra were conducted by BEM simulations using the MNPBEM toolbox.^50^ Maxwell’s equations are solved under boundary conditions, given by the experimentally determined structure parameters. For gold, a tabulated dielectric function in the energy range from 0.1 to 6 eV was used.^66^ To account for substrate effects, the structure was embedded in an effective medium with a dielectric constant of 2.8. The local excitation of the structures was established via a beam of 200 keV electrons with a width of 0.2 nm. BEM simulations were performed in the energy range between 0.125 and 2.3 eV with mesh 350, resulting in steps of around 6 meV.

### CL Measurements

CL measurements were performed using a ZEISS SIGMA field emission SEM operated at an acceleration voltage of 20 kV and a resulting probe current of 10.4 nA. The optical spectroscopy capabilities are achieved by an implemented parabolic mirror that is positioned below the pole piece with the specimen in its focal point and guides the emitted far-field radiation of the excited sample outside the microscope chamber. The electron beam passes through a hole in the mirror with a diameter of 0.6 mm. The emitted light is analyzed in the attached delmic SPARC Spectral CL system. In the spectroscopic mode, the emission is detected per spatial pixel of the scanned area of interest either as a dispersed spectrum or filtered for certain wavelength windows with a high SNR. Spectra were acquired with an exposure time of 200 ms, a 220 μm entrance slit aperture, and a grating with 150 l/mm. In a second configuration, the detected signal strength is related to its emission direction for a fixed excitation position and wavelength window with a bandwidth of 50 nm. These angle-resolved measurements can be combined with linear polarizers and a quarter wave plate (QWP) for full characterization of the polarization state via Stokes parameters. These measurements were performed with an exposure time of 30 s for each filter configuration. The light emitted from the structures is collected via a parabolic mirror and directed to the analyzing path, which constitutes a bandpass filter, a quarter-wave-plate, and a linear polarizer, and directly projected onto the CCD camera. The obtained spatially resolved signal is transferred to a three-dimensional coordinate system via geometrical considerations, relating each detected intensity to its emission direction. Perturbing effects on the polarization state due to the reflection at the curved aluminum surface of the mirror are considered via the Fresnel formalism. Using the Mueller matrix, detected polarization maps are transformed back to the initial state, as emitted by the sample (Figure S12).^37^

### CL Simulations

Numerical simulations regarding CL emission were conducted using the COMSOL Multiphysics software package with its radiofrequency (RF) toolbox. Maxwell’s equations are solved in the frequency-domain by using the finite-element method and in real space of a three-dimensional simulation domain, based on the experimentally determined geometries of the structures. To simulate the electron beam, we employ an oscillating “edge current” along a straight line, which serves as the source for the electromagnetic field in the system. The current is expressed by , where *v*_*e*_ is the speed of the moving electron along the *z*-axis. CL spectra are obtained by integrating the Poynting vector of the three coordination components of the emitted light across the entire top surface of the hemisphere. For the *S*3 parameter, we utilized the transversal components of the electric field and their vector distribution at each spatial point. In this calculation, we applied the formula *S*3 = *i*(*E*_∥_*E*_⊥_^*^ – *E*_⊥_*E*_∥_^*^) at the far-field components of the electric field projected on the top and bottom hemispheres.

## Supporting Information Available

Rotating model of the reconstructed left-handed nanohelix (MPG)
Rotating model of the reconstructed right-handed nanohelix (MPG)
Animated model, showing the simulated dynamic evolution of the rotating electric field, induced through electron excitation (AVI)
Additional figures and information about the sample fabrication process, the three-dimensional tomographic reconstruction of individual nanohelices, additional experimental and simulated spectral data about gold nanorods and nanohelices, spectroscopic, angle-resolved, and polarimetric CL measurements and simulations, and technical information about experimental CL measurements (PDF)

## Supplementary Material

nn3c09336_si_001.mpg

nn3c09336_si_002.mpg

nn3c09336_si_003.avi
