# Supplementary material for: Electron Beam Induced Circularly Polarized Light Emission of Chiral Gold Nanohelices
Source: ACS Nano. 2023 Nov 22;17(24):25496–506. doi: 10.1021/acsnano.3c09336 (PMC10753880; doi:10.1021/acsnano.3c09336)
Supplement: Supplementary file 4 — nn3c09336_si_004.pdf [file nn3c09336_si_004.pdf]

Supplementary Information

Electron Beam Induced Circularly Polarized Light Emission of Chiral Gold Nanohelices

Robin Lingstädt[1,*], Fatemeh Davoodi[2,*], Kenan Elibol[1], Masoud Taleb[2], Hyunah Kwon[3,4], Peer Fischer[3,4], Nahid Talebi[2,5] and Peter A. van Aken[1]

[1] Max Planck Institute for Solid State Research, Stuttgart, 70569, Germany
[2] Institute of Experimental and Applied Physics, Christian Albrechts University, Kiel, 24118, Germany
[3] Max Planck Institute for Medical Research, Heidelberg, 69120, Germany
[4] Institute for Molecular Systems Engineering and Advanced Materials, Heidelberg University, Heidelberg, 69120, Germany
[5] Kiel Nano, Surface and Interface Science KiNSIS, Christian Albrechts University, Kiel, 24118, Germany

* Corresponding authors r.lingstaedt@fkf.mpg.de
davoodi@physik.uni-kiel.de

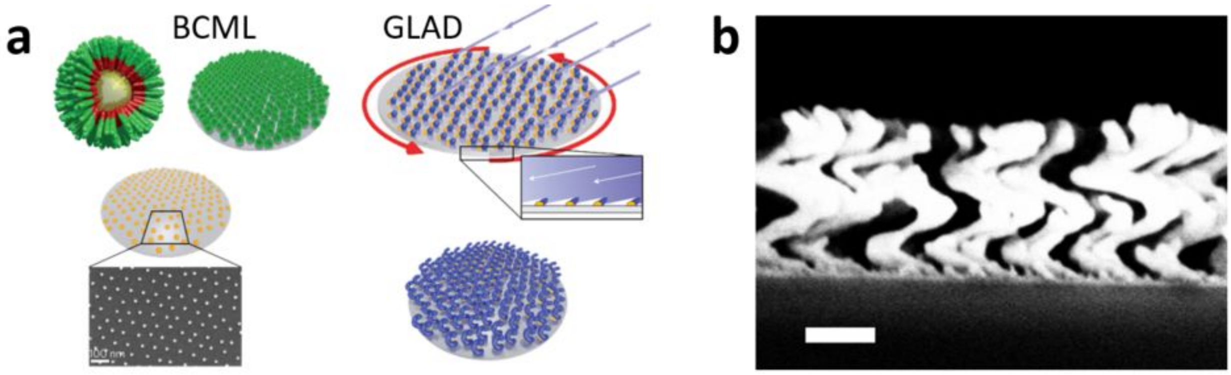

Figure S1 Sample fabrication.
(a) Gold nanohelices were produced via a combination of Block Copolymer Micelle Lithography (BCML) and Glancing Angle Deposition (GLAD). The images are reproduced or adapted with permission from [1,2]. Copyright 2013, Nature Materials and 2014, Nanoscale. (b) SEM image of the grown nanohelices. The scale bar is 100 nm.

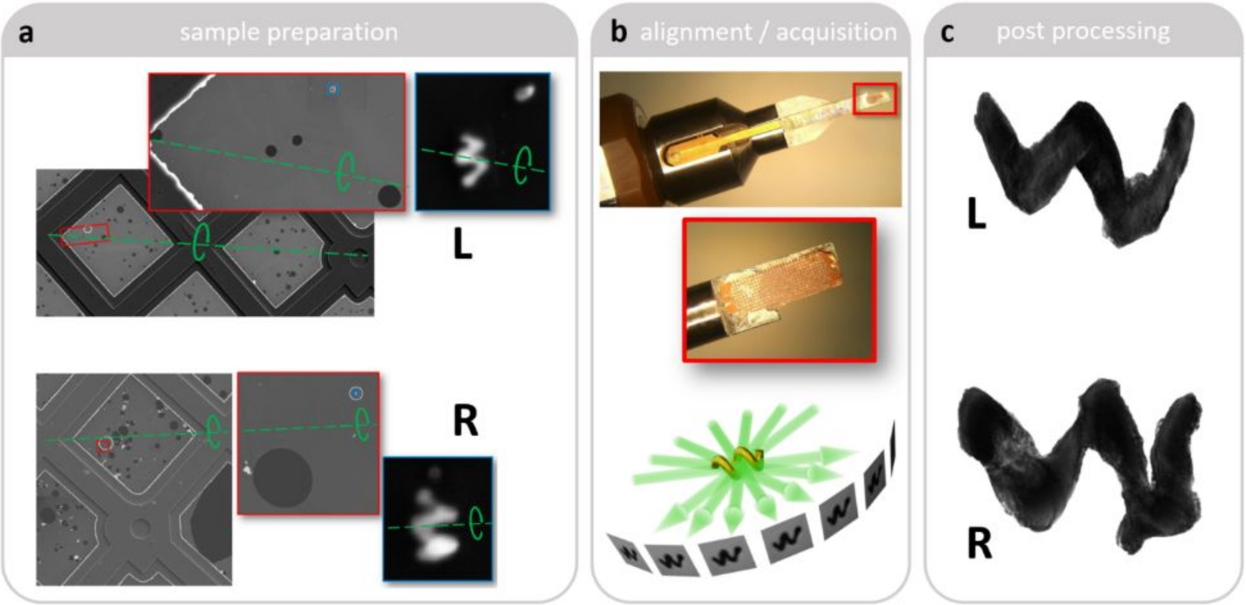

Figure S2 **Electron tomography**.

(a) Sample grids containing the particles of interest for electron tomography are cut in a way that shadowing effects during the tilt series acquisition around the rotation axis (green dashed line) are minimized. (b) The prepared grid is mounted onto the tip of the tomography holder that allows for tilt angles between +/- 70 degrees inside the electron microscope. (c) Reconstructed three-dimensional nanohelices show the expected handedness of the chiral structures. Movies of the rotating three-dimensional reconstructions are available online.

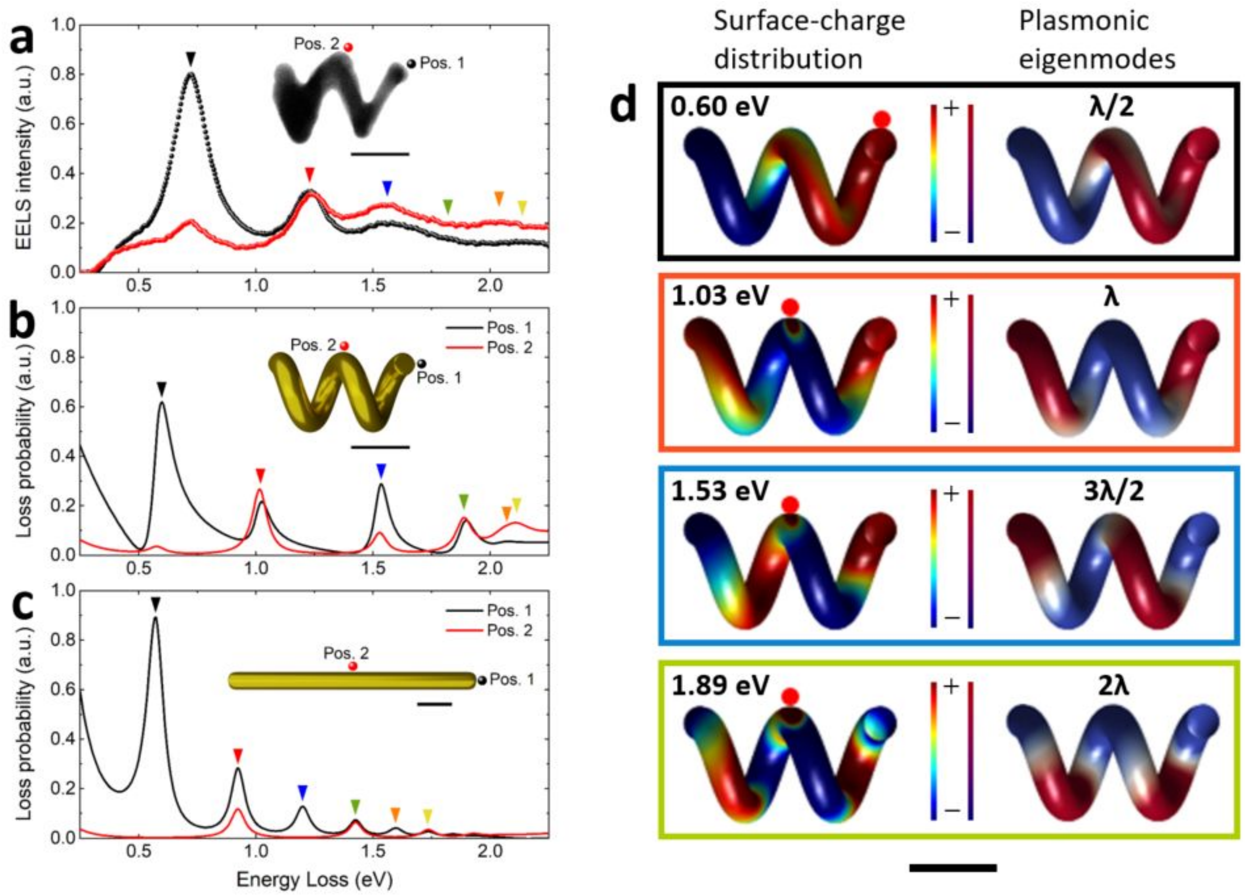

Figure S3 LSPRs of a right-handed nanohelix.

(a) Experimental EEL spectra of a right-handed nanohelix, being excited at positions 1 at the end of the structure (black curve) and 2 at the center winding (red curve) in an aloof configuration, as marked in the inset graphic. A strong absorption peak at the lowest resonance energy around 0.7 eV is observed at the end of the structure, whereas the second resonance around 1.2 eV and all higher-order resonances are stronger at the center. (b) Simulated EEL spectra obtained via the BEM. (c) A straight nanorod with identical thickness and center path length as the helix reveals that the observed resonances originate from “antenna modes” of multiple orders. While the absorption peak of the fundamental dipolar mode is located at the same energy around 0.6 eV like the one of the helix, all higher-order modes appear redshifted. (d) Simulated surface-charge distributions and underlying plasmonic eigenmodes computed for the depicted resonance energies. The excitation position of the electron beam is marked with a red dot. The scale bars are 100 nm.

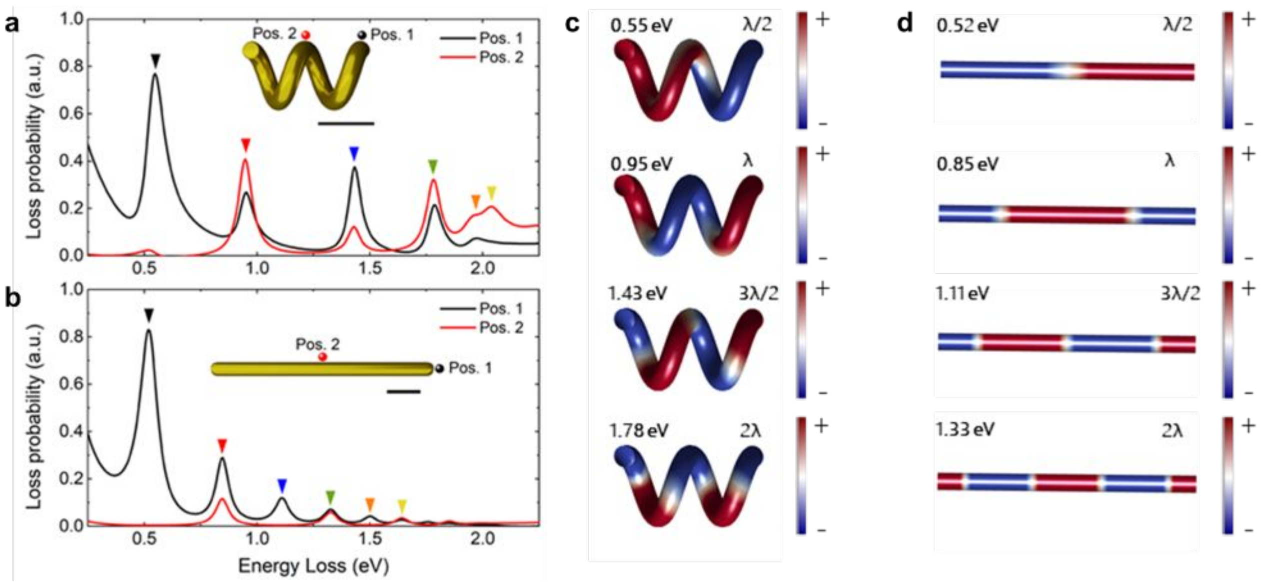

Figure S4 Comparison with antenna modes of gold nanorods.
(a) Simulated EEL spectra of a left handed helix. (b) Simulated EEL spectra of a straight nanorod with identical thickness and center path length as the helix reveals that the observed resonances originate from "antenna modes" of multiple orders. While the spectral peak of the fundamental dipolar mode is located at the same energy around 0.5 eV like the one of the helix, all higher-order modes appear redshifted. (c,d) Plasmonic eigenmodes of a left-handed helix and a nanorod of the same size.

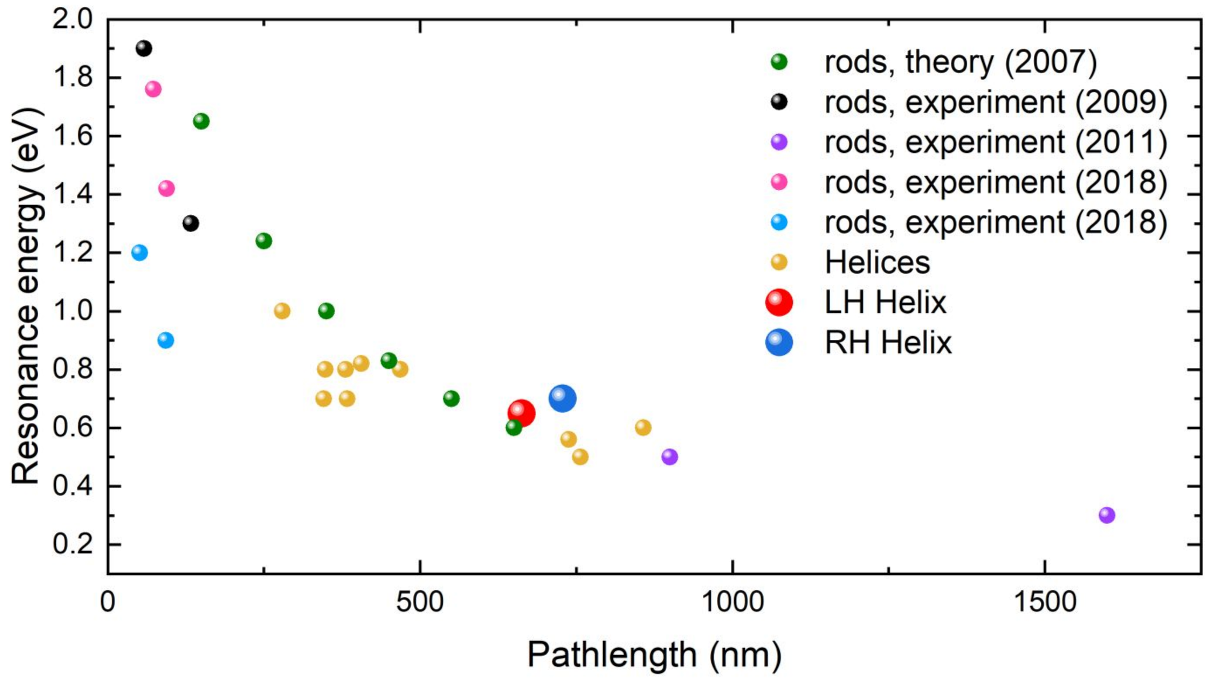

Figure S5 Dipolar resonances of gold nanorods and helices.
Resonance energies are obtained from [3–7]. The labels "LH Helix" in red color and "RH Helix" in blue color mark the values for the helical samples, which are discussed in the main document. For nanorods, the "Pathlength" is equal to the length of the rod.

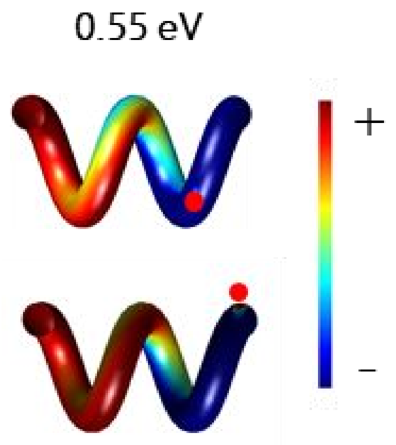

Figure S6 Plasmonic modes for general excitation positions.
Simulated surface-charge distributions computed for a left-handed nanohelix at the depicted resonance energy. The excitation position of the electron beam is marked with a red dot. At a more general electron beam position along the helical winding (upper graph), the plasmonic mode, as discussed in the main manuscript (lower graph), remains clearly visible.

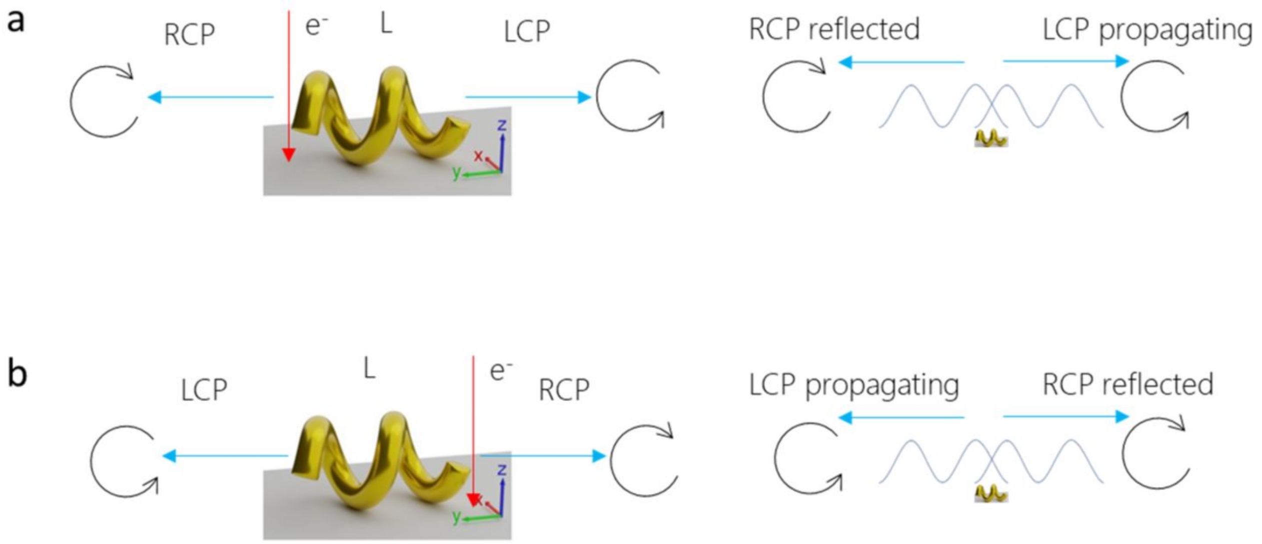

Figure S7 LCP and RCP light emission and reflection from left-handed helix. Schematic showing the mechanisms inducing emission of the LCP and RCP light from the left-handed helix exited from its left end (a) and right end (b). Here, the wavelengths of the LCP and RCP light of 800 nm is approximately four times larger than the length of the helix (~200 nm).

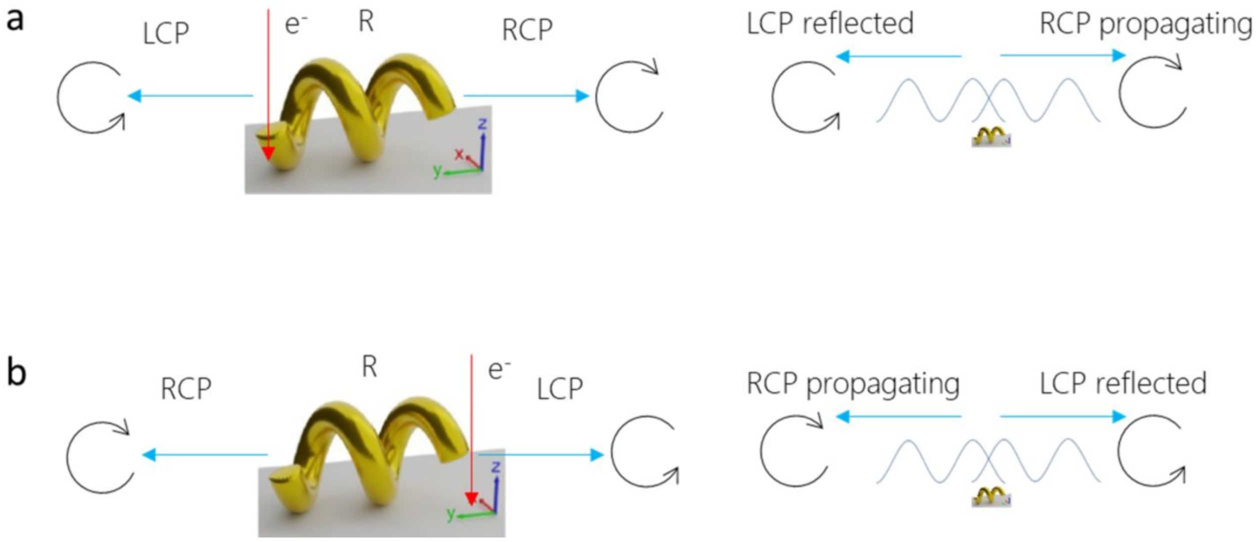

Figure S8 LCP and RCP light emission and reflection from right-handed helix. Schematic showing the mechanisms inducing emission of the LCP and RCP light from the right-handed helix exited from its (a) left end and (b) right end. Here, the wavelengths of the LCP and RCP light (800 nm) are approximately four times larger than the length of the helix (~200 nm).

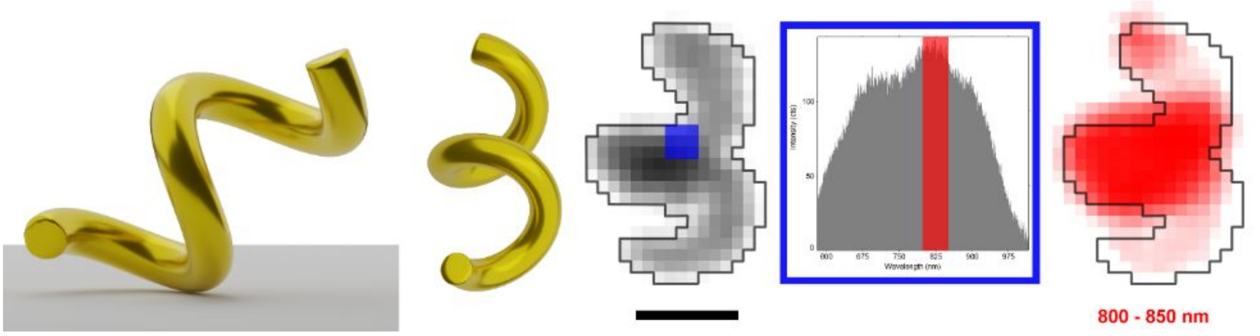

Figure S9 Spectral response of a tilted structure.
Side view and top view of a tilted nanohelix. Its orientation does not significantly affect the spectral response, despite of a small perspective overlay in the excitation efficiency map.

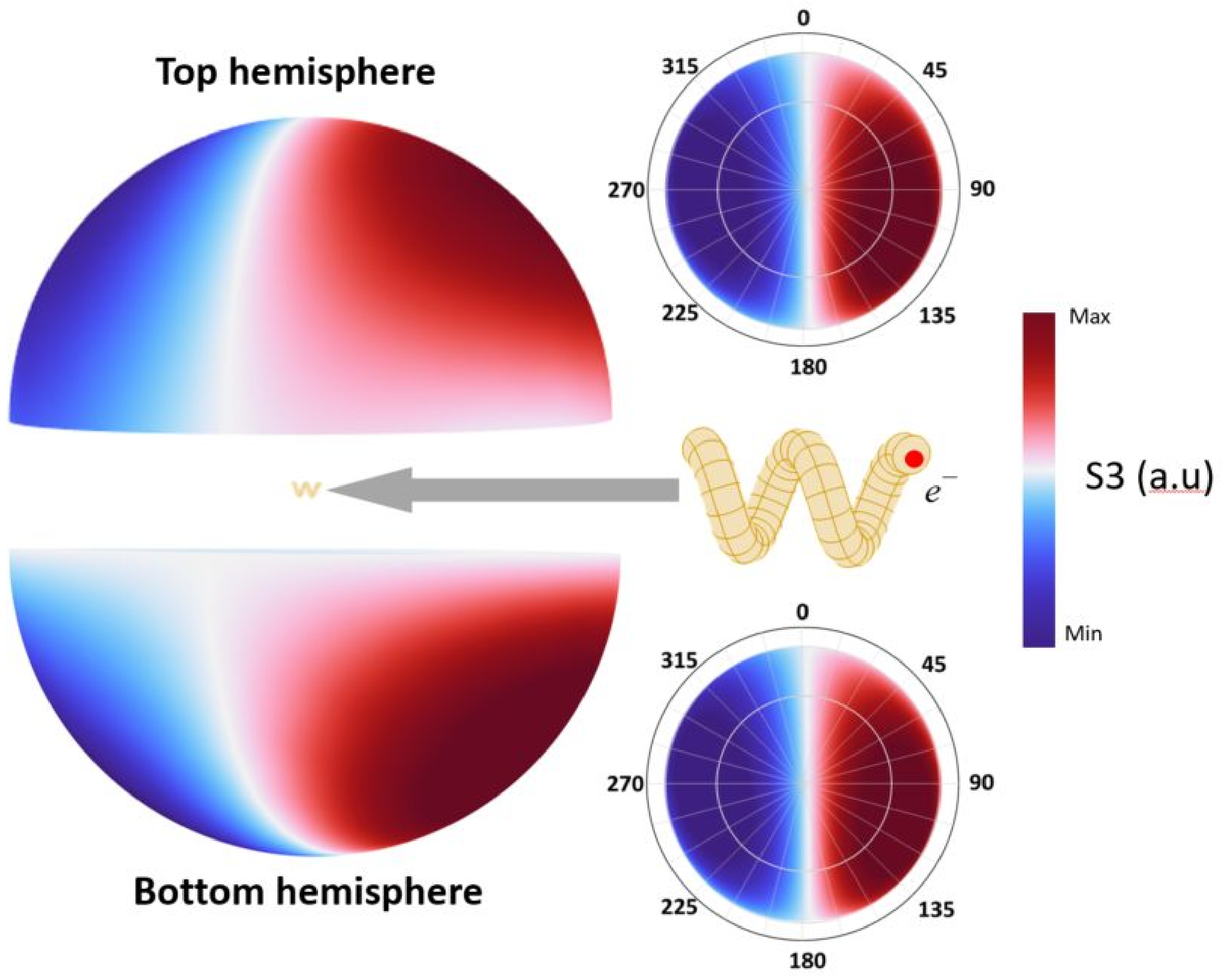

Figure S10 Angle-resolved polarimetry simulation for the bottom hemisphere.
The dichroic signal of LCP and RCP light detected at the top and bottom hemispheres results in similar intensity distributions.

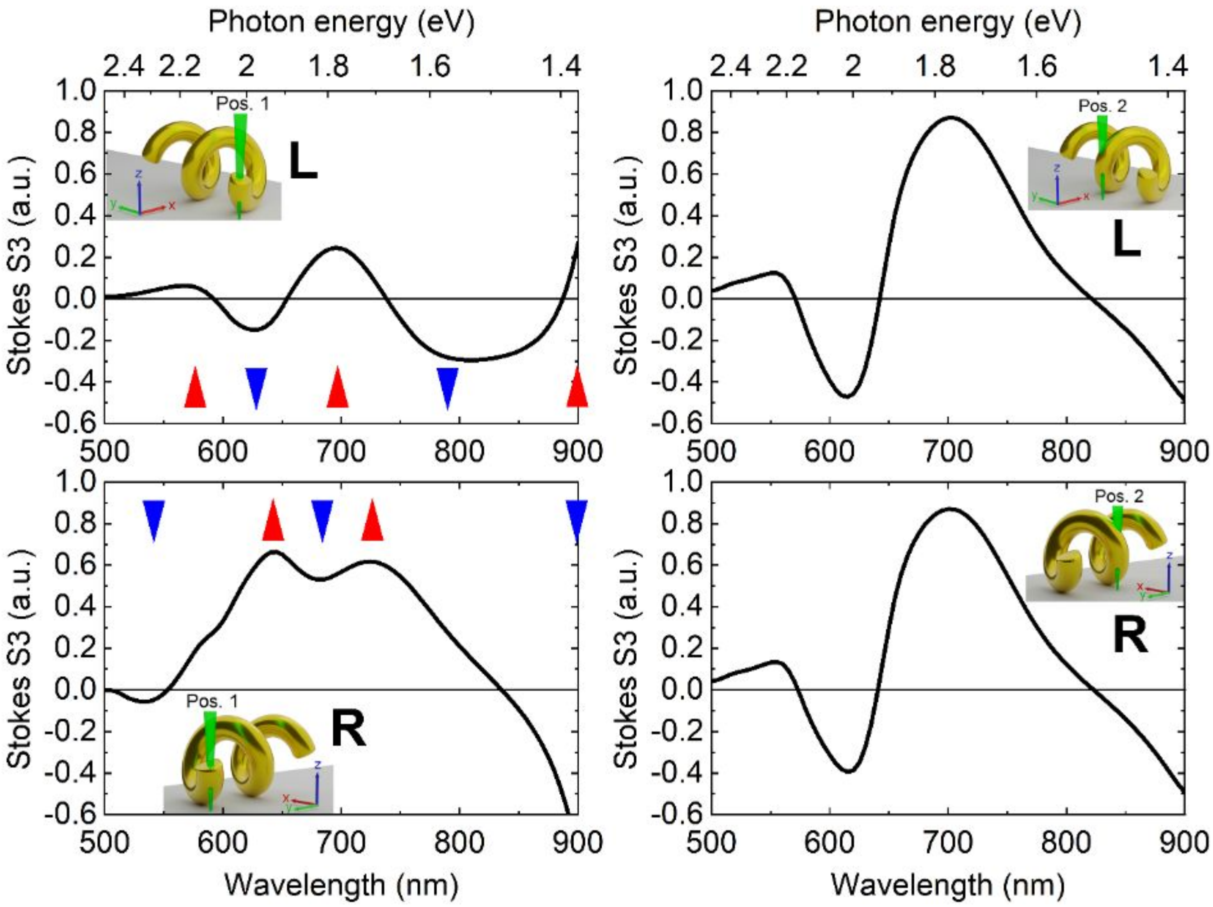

Figure S11 Stokes S3 for left- and right-handed nanohelices with identical dimensions.
Stokes S3 parameters for the left- and right-handed helices excited from different positions.

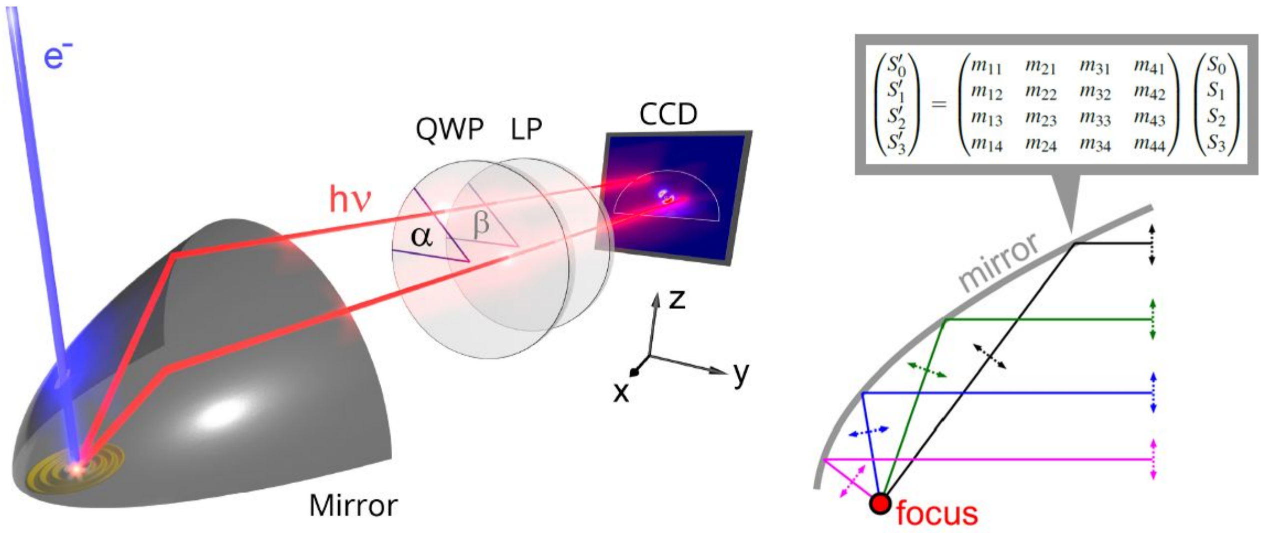

Figure S12 Angle-resolved polarimetry measurements.
Images of the parabolic mirror contain spatially resolved intensity maps that are geometrically related to emission directions. The contributing emission, filtered for a certain wavelength range, is analyzed via a combination of a linear polarizer (LP) and quarter wave plate (QWP) (left graphic). The perturbing effect of the aluminum mirror on the polarization states is corrected via Mueller matrix calculations (right graphic). Images are reproduced or adapted with permission from [8,9]. Copyright 2021 and 2019, delmic.

References

1. Mark, A. G., Gibbs, J. G., Lee, T. C. & Fischer, P. Hybrid nanocolloids with programmed three-dimensional shape and material composition. *Nat. Mater.* **12**, 802–807 (2013).

2. Gibbs, J. G. *et al.* Nanohelices by shadow growth. *Nanoscale* **6**, 9457–9466 (2014).

3. Bryant, G. W., García de Abajo, F. J. & Aizpurua, J. Mapping the Plasmon Resonances of Metallic Nanoantennas. *Nano Lett.* **8**, 631–636 (2008).

4. N'Gom, M. *et al.* Electron-beam mapping of plasmon resonances in electromagnetically interacting gold nanorods. *Phys. Rev. B* **80**, 113411 (2009).

5. Alber, I. *et al.* Visualization of Multipolar Longitudinal and Transversal Surface Plasmon Modes in Nanowire Dimers. *ACS Nano* **5**, 9845–9853 (2011).

6. Cai, Y. Y. *et al.* Photoluminescence of Gold Nanorods: Purcell Effect Enhanced Emission from Hot Carriers. *ACS Nano* **12**, 976–985 (2018).

7. Kobylko, M. *et al.* Localized Plasmonic Resonances of Prolate Nanoparticles in a Symmetric Environment: Experimental Verification of the Accuracy of Numerical and Analytical Models. *Phys. Rev. Appl.* **9**, 064038 (2018).

8. delmic SPARC Spectral technical note on "Polarization-Filtered Cathodoluminescence Imaging", Version V01-01, 2021-12-11, *delmic* (2021)

9. delmic SPARC Spectral "manual", generic version 1.22, January 2019, *delmic* (2019)
